# Supplementary material for: A lung-selective delivery of mRNA encoding broadly neutralizing antibody against SARS-CoV-2 infection
Source: Nat Commun. 2023 Dec 5;14:8042. doi: 10.1038/s41467-023-43798-8 (PMC10697968; doi:10.1038/s41467-023-43798-8)
Supplement: Supplementary file 1 — Supplementary Information [file 41467_2023_43798_MOESM1_ESM.pdf]

A lung-selective delivery of mRNA encoding broadly neutralizing antibody against SARS-CoV-2 infection

Wanbo Tai, Kai Yang, Yubin Liu, Ruofan Li, Shengyong Feng, Benjie Chai, Xinyu Zhuang, Shaolong Qi, Huicheng Shi, Zhida Liu, Jiaqi Lei, Enhao Ma, Weixiao Wang, Chongyu Tian, Ting Le, Jinyong Wang, Yunfeng Chen, Mingyao Tian, Ye Xiang, Guocan Yu, Gong Cheng

Corresponding to

gongcheng@mail.tsinghua.edu.cn,

guocanyu@mail.tsinghua.edu.cn,

yxiang@mail.tsinghua.edu.cn,

klwklw@126.com.

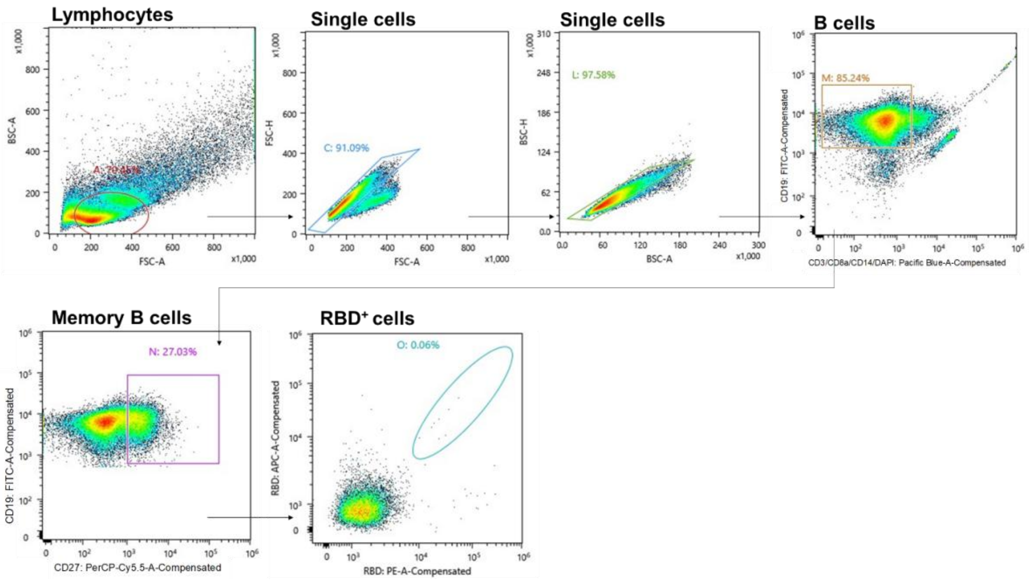

Supplementary Fig. 1 Isolation of SARS-CoV-2 RBD-specific antibodies through fluorescence-activated cell sorting (FACS). Single $CD3^-CD8^-CD14^-CD19^+CD27^+$ cells were gated, from which RBD-PE^+ and RBD^-APC^+ cells were sorted. FSC-A: forward scatter area. FSC-H: forward scatter height. BSC-A: back scatter area. BSC-H: back scatter height.

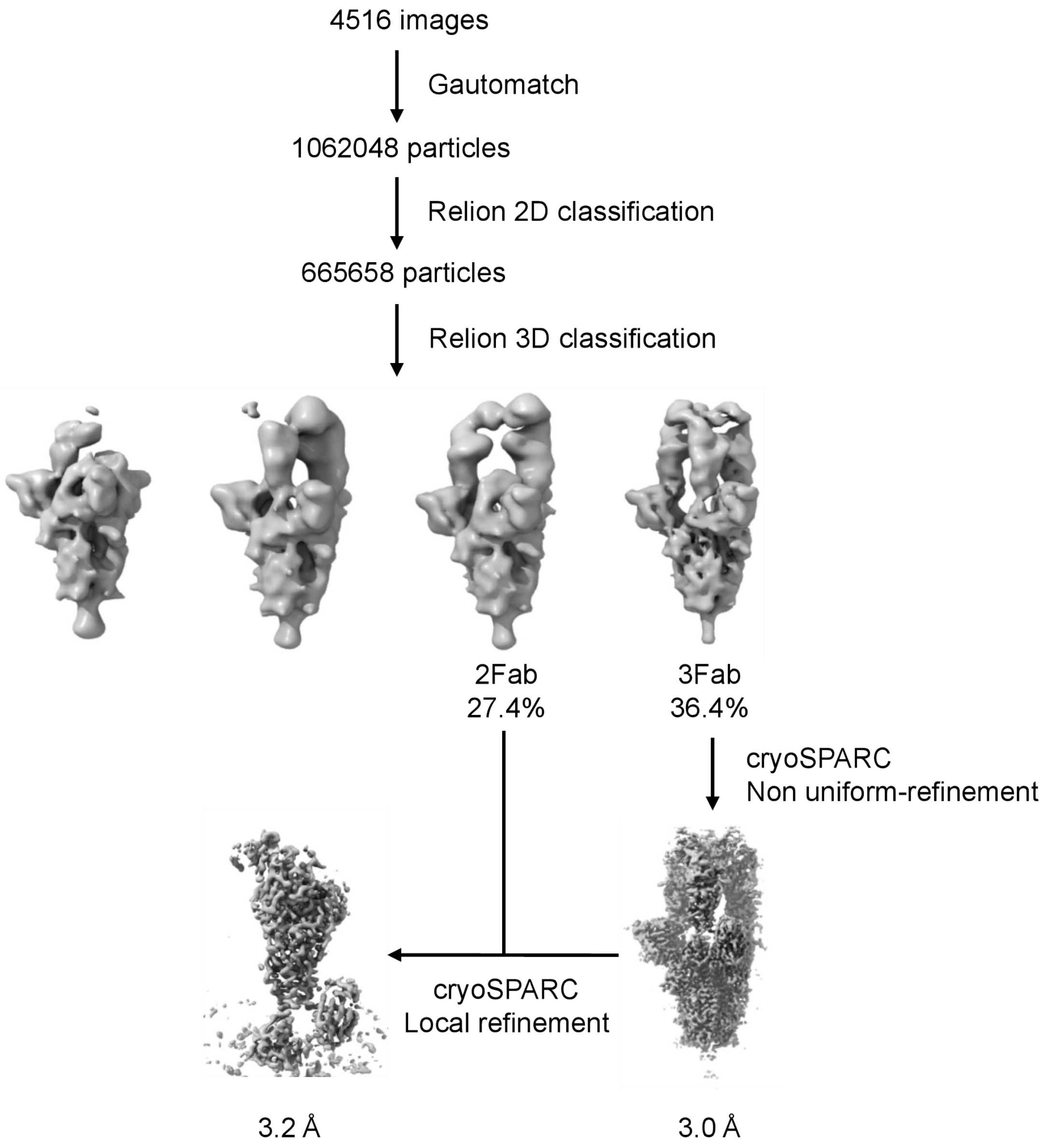

Supplementary Fig. 2 The workflow of cryo-EM data processing of 8-9D fabs in complex with Omicron BA.5 S trimer.

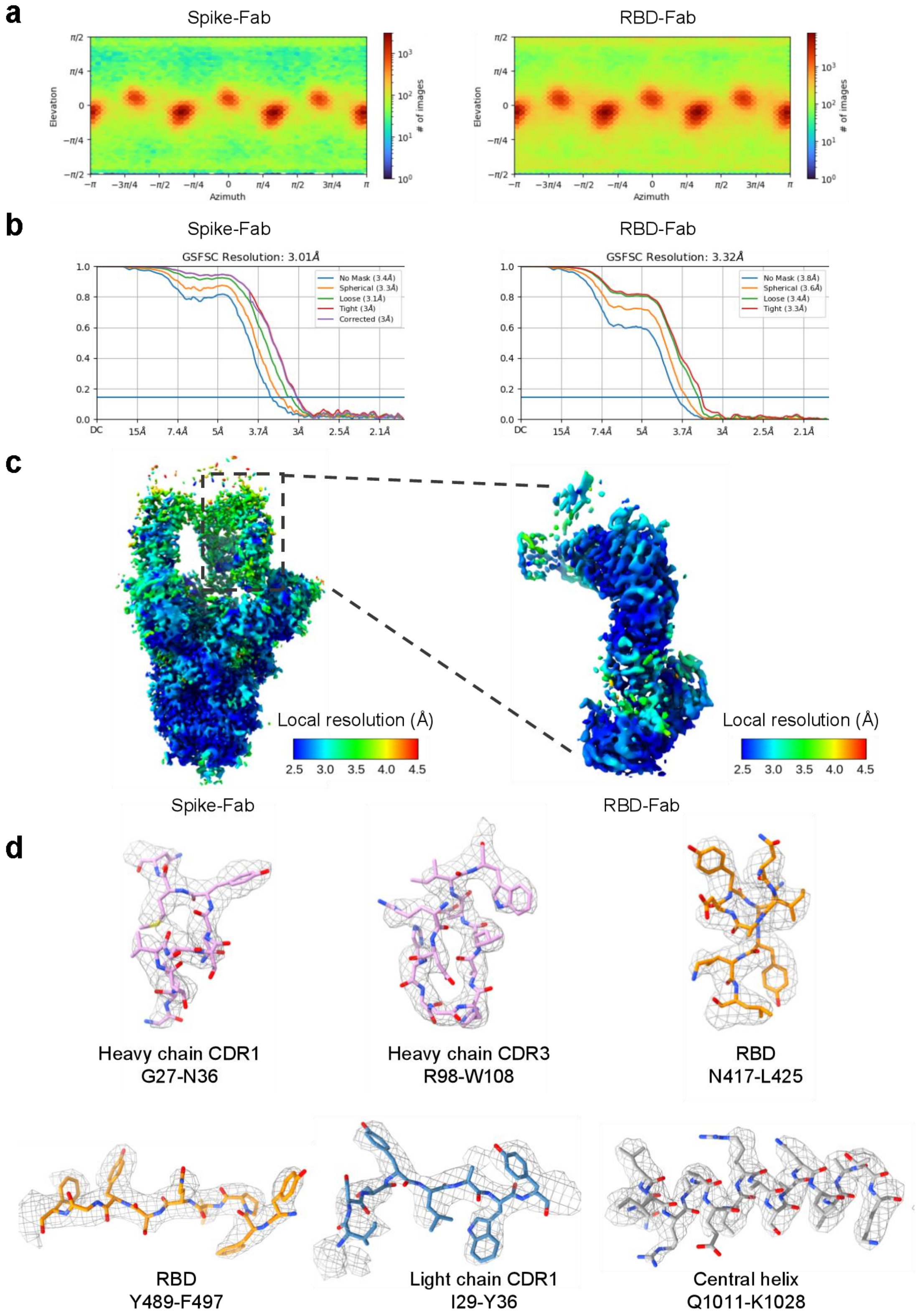

Supplementary Fig. 3 Angular distributions, FSC curves and local resolution maps of reconstructed 8-9D-spike complex. a, The angular distribution of particles used for the global (left) and local (right) refinement. **b**, Gold-standard Fourier shell correlation (FSC) curves of reconstructed global (left) and local (right) maps. The FSC curves were calculated

by cryoSPARC with the 0.143 threshold. **c,** Local resolution maps were calculated with half maps from global (left) and local (right) refinement jobs by using cryoSPARC. **d,** Representative densities of the Omicron BA.5 spike in complex with 8-9D Fab**.** The deepEMhancer filtered maps are drawn at a contouring level of 0.197 $e^-/Å^3$. The atomic coordinates are shown in sticks with O and N atoms colored red and dark blue, respectively. The heavy chain and light chain of 8-9D are colored pink and blue, respectively. The RBD and central helix (CH) of spike is colored orange and gray, respectively.

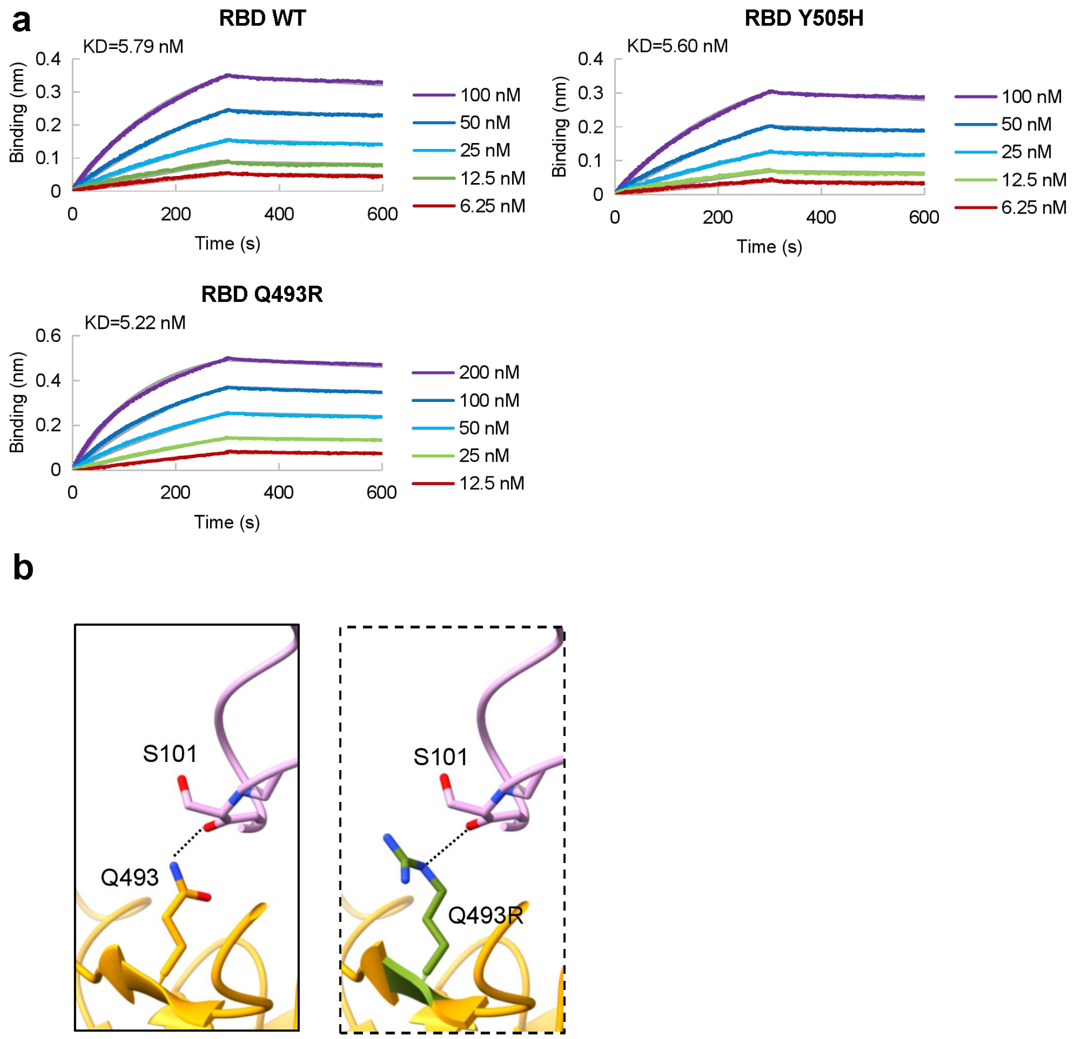

Supplementary Fig. 4 BLI analysis of the binding of mAb 8-9D to wild type RBD or RBD mutants. a, The association and disassociation curves of mAb 8-9D to the wild type RBD or RBD mutants measured with BLI. **b,** Ribbon and stick diagrams comparing the hydrogen bond mediated by N493 (in solid rectangle) and modeled interactions between 8-9D and RBD with the Q493R mutation (in dashed rectangle).

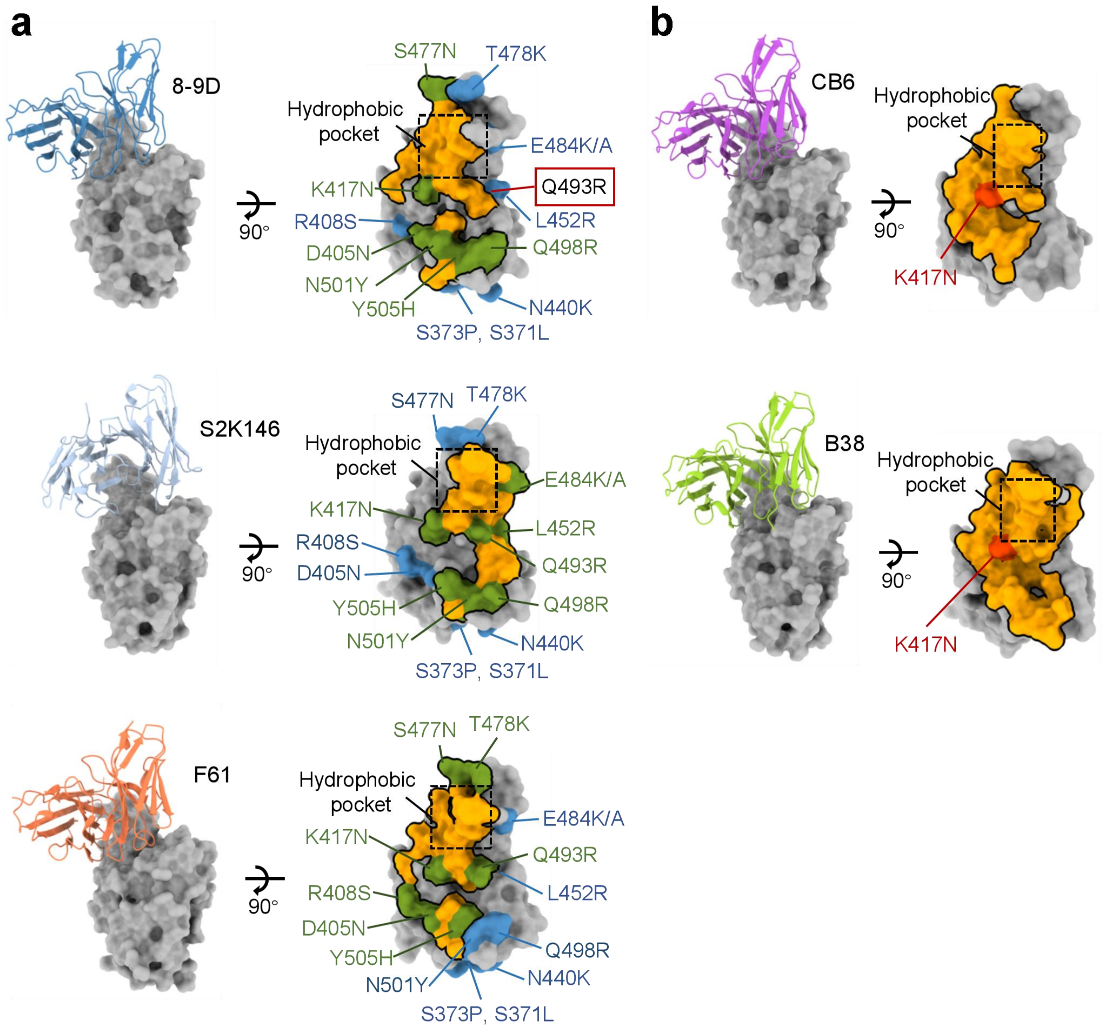

Supplementary Fig. 5 Comparison of antibodies with similar epitopes as 8-9D. a, Left panel, atomic models showing Fabs binding to RBDs. 8-9D is colored dark blue, S2K146 is colored light blue, F61 is colored coral. Right panel, mutations mapping on each epitope of the corresponding antibody. Each epitope is outlined in black, residues of each epitope are colored orange, mutated residues outside each epitope are colored blue, mutations inside each epitope still interact with the corresponding antibody are colored green. The mutation Q493R that impairs 8-9D neutralizing activity is boxed in red outline. **b,** Left panel, atomic models showing Fabs binding to RBDs. CB6 is colored purple and B38 is colored green. The surface of RBDs is colored dark gray. Right panel, Mutations mapping on CB6 and B38 epitopes. Each epitope is outlined in black, residues of each epitope are colored orange. K417N mutation is colored red.

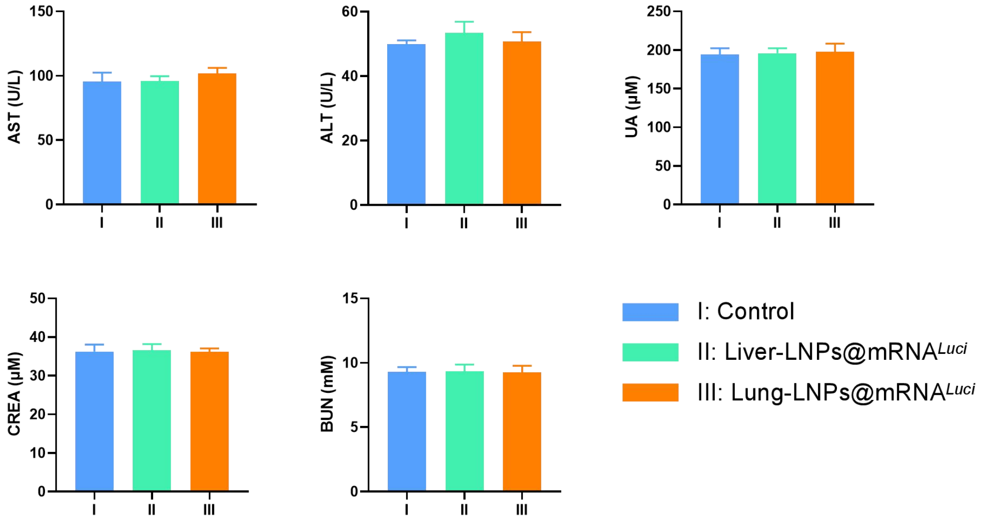

Supplementary Fig. 6 Evaluation of liver and renal function. The liver and renal function indexes including alanine aminotransferase (ALT), aspartate aminotransferase (AST), uric acid (UA), blood urea nitrogen (BUN), and creatinine (CREA) from mice after administration of Liver-LNPs@mRNA^{Luci}, Lung-LNPs@mRNA^{Luci} or PBS (Control) (n = 4 biologically independent samples). Data are presented as mean ± SD. Source data are provided as a Source Data file.

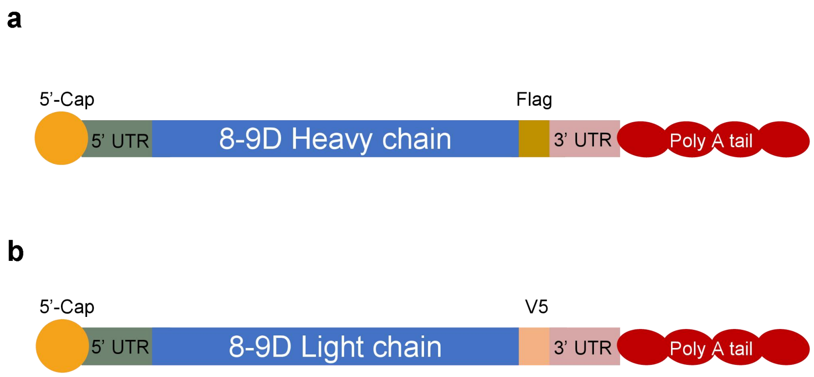

Supplementary Fig. 7 Schematic diagram of 8-9D heavy and light chain mRNA. To construct and synthesize the 8-9D mRNA, optimized 5'-UTR and 3'-UTR, poly A tail (104 bp) and Cap1 were added to the mRNA sequence. The Flag tag and V5 tag were included at the carboxy terminus of the heavy chain (**a**) and light chain (**b**) respectively.

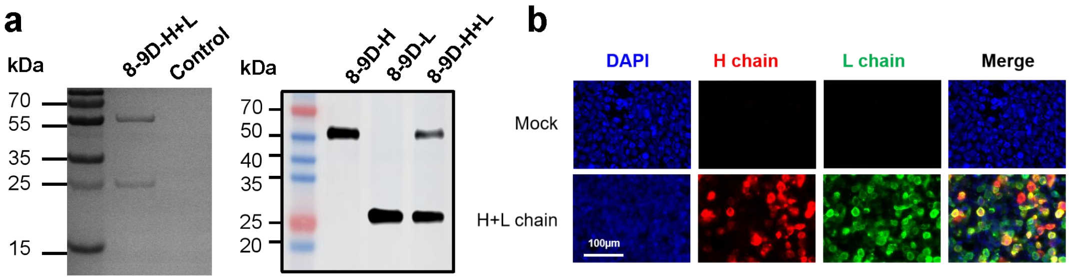

Supplementary Fig. 8 In vitro expression validation of 8-9D mRNA. a,b, 8-9D mRNA expression *in vitro*. HEK293T cells were transfected with 8-9D mRNA. At 48 hours post transfection, the supernatant was harvested for SDS–PAGE (**a**, left) and Western blotting (**b**, right), and the cells were subjected to immunofluorescence staining (**b**).

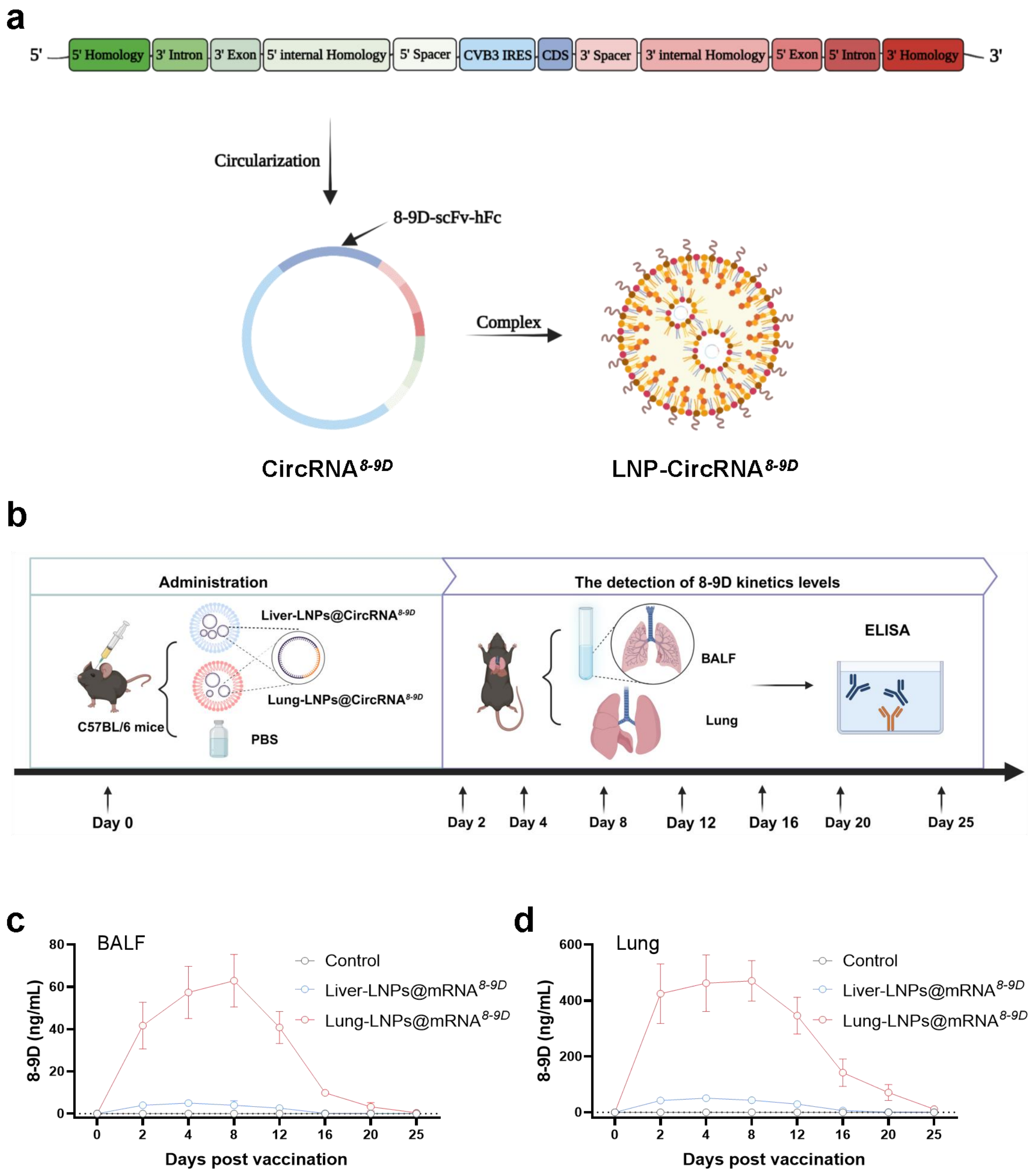

Supplementary Fig. 9 Circular 8-9D mRNA elongates the half-life. a. The design of circular 8-9D mRNA. **b.** Schematic for distribution and kinetics analysis of circular 8-9D mRNA after injection. **c,d.** 8-9D kinetics levels in BALF (**c**) and lungs (**d**) were further measured for the samples (n = 3) collected on Day 2, 4, 8,12, 16, 20 and 25 post injection. Data are presented as mean ± SD of the experiments. Source data are provided as a Source Data file.

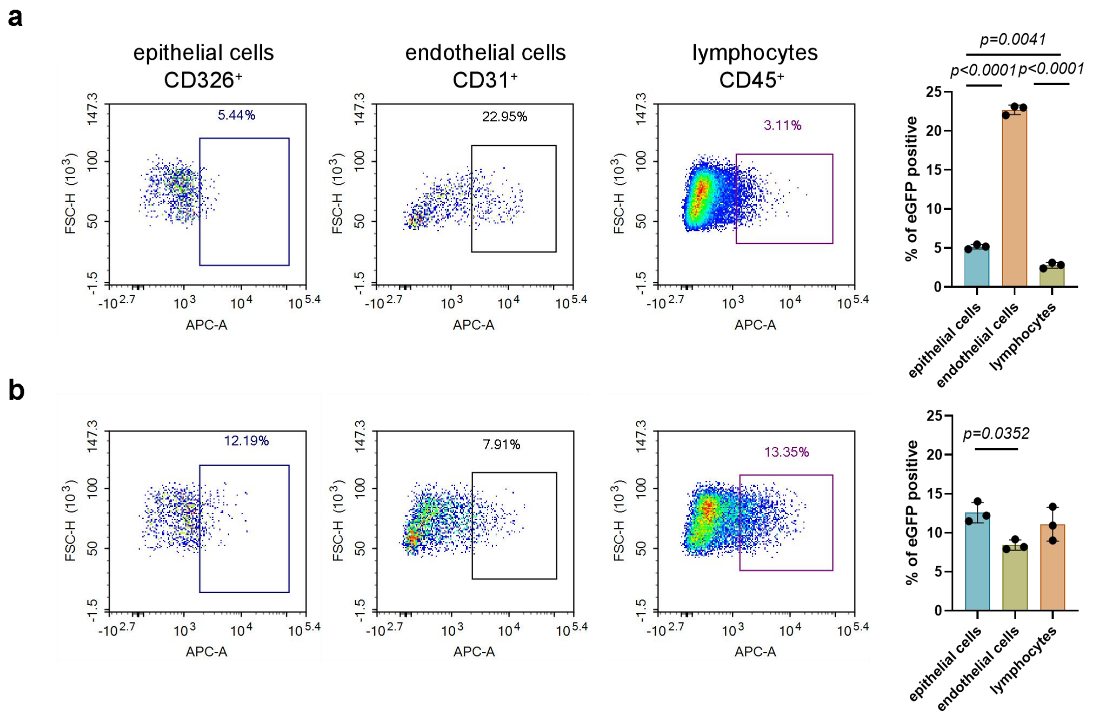

Supplementary Fig. 10 Cell types being transfected in lungs by the lung targeting LNPs. Mice (n = 3) intravenously injected Lung-LNPs@mRNA^{eGFP} were sacrificed and Lungs were harvested and analyzed for the eGFP signal by flow cytometry. The transfected percentage of epithelial cells, endothelial cells and lymphocytes were quantitatively analyzed within lungs at 2 hours (**a**) and 6 hours (**b**) post injection. Data are presented as mean ± SD of the experiments. Source data are provided as a Source Data file.

a

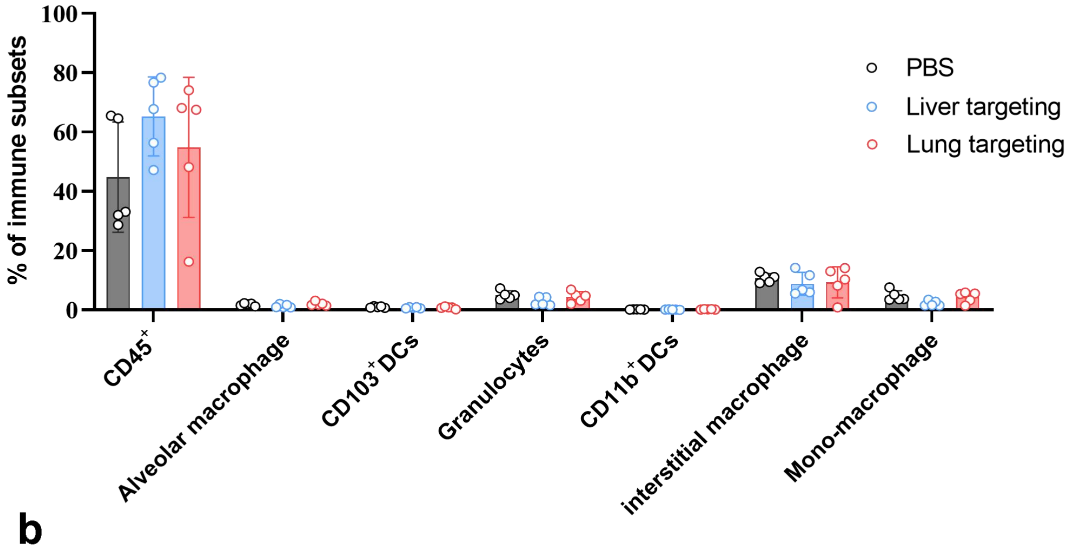

b

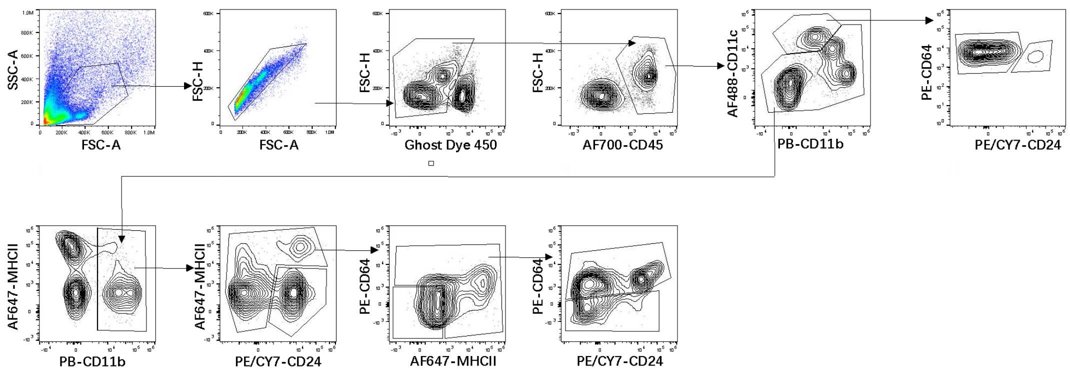

Supplementary Fig. 11 Pulmonary immune cell subset of lungs. a, The relative distribution of pulmonary immune cell subsets in lungs from Liver-LNPs@mRNA$^{8\text{-}9D}$ or Lung-LNPs@mRNA$^{8\text{-}9D}$ or PBS-treated mice (6 hours post injection, n = 5 mice). **b,** Gating strategy of flow cytometry for pulmonary immune cell subsets. Data are presented as mean ± SD of the experiments. Source data are provided as a Source Data file.

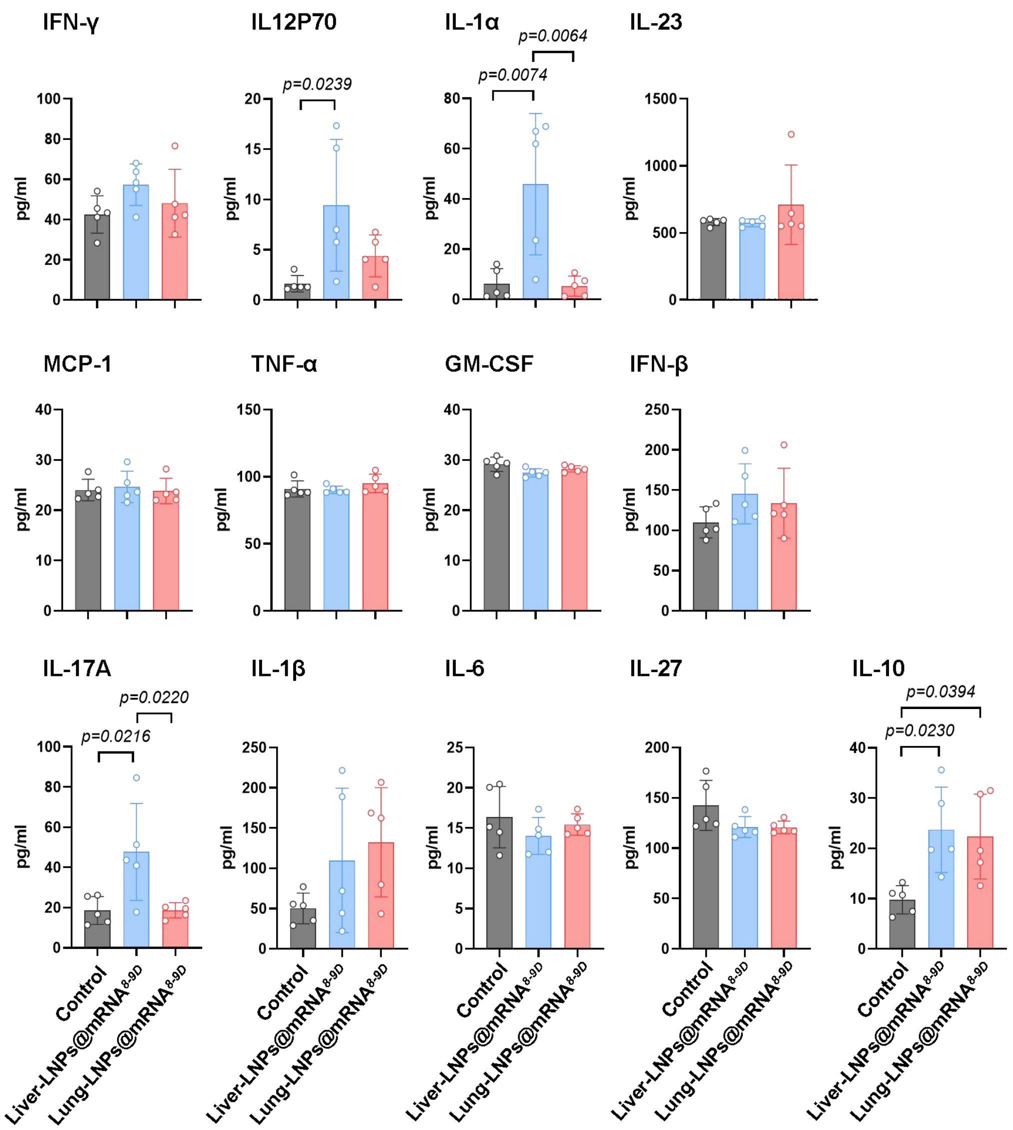

Supplementary Fig. 12 Inflammatory cytokine responses. Serum samples collected from Liver-LNPs@mRNA$^{8\text{-}9D}$, Lung-LNPs@mRNA$^{8\text{-}9D}$ or PBS-treated (Control) mice at 6 hours post injection were screened for the inflammatory cytokine responses. Statistical analyses on datasets were performed by one-way ANOVA with Tukey's post hoc test. Data are presented as mean ± SD of the experiments. Source data are provided as a Source Data file.

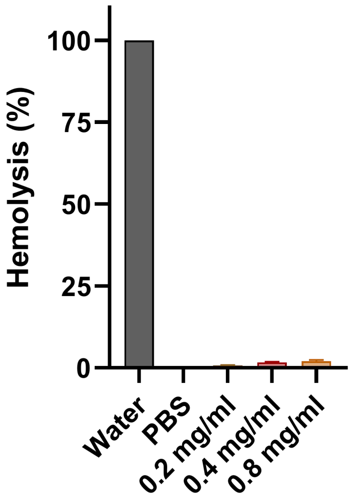

Supplementary Fig. 13 Hemolysis induced by Lung-LNPs. Erythrocytes suspension was incubated for 1 hour with the nanoparticles at concentrations up to 20% (v/v). The absorbance of the supernatant was measured at 415 nm. The percentages of nanoparticles correspond to 0.2, 0.4 and 0.8 mg/ml of lipids, respectively. The hemolysis induced by water was selected as 100% and was used to calculate the hemolysis percentage induced by nanoparticles. Data are presented as mean ± SD of the experiments. Source data are provided as a Source Data file.

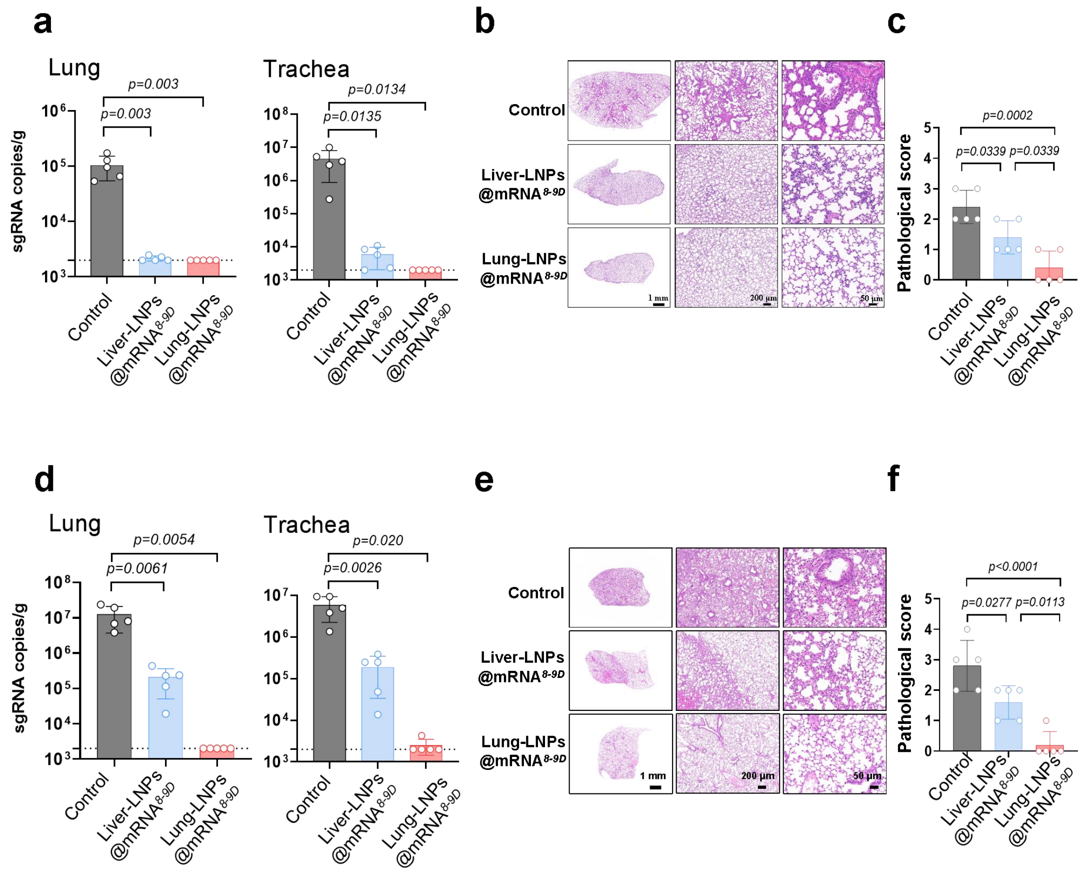

Supplementary Fig. 14 8-9D mRNA protective efficacy in SARS-CoV-2 animal model. **a–c**, 8-9D mRNA protective efficacy as prophylaxis, the K18-hACE2 mice (n = 5) were i.v. injected with 5 μg of Liver-LNPs@mRNA$^{8\text{-}9D}$ or Lung-LNPs@mRNA$^{8\text{-}9D}$. Twenty-four hours post LNP injection, the mice were challenged with 2 × 10^4 $TCID_{50}$ Omicron BA.2 variant. Four days post challenge, the mice were euthanized, and the lungs and tracheae were collected for viral load analysis by qRT–PCR (**a**). The lungs were fixed for pathology evaluation (**b**), and pathological scores (**c**) were determined for significant comparison. PBS-injected mice were set as control. **d–f**, 8-9D mRNA protective efficacy as treatment. The mice were first anesthetized and intranasally inoculated with 2 × 10^4 $TCID_{50}$ of authentic SARS-CoV-2 Omicron BA2 variant, and after 24 hours, the mice were intravenously administered one dose of 5 μg of Liver-LNPs@mRNA$^{8\text{-}9D}$ or Lung-LNPs@mRNA$^{8\text{-}9D}$, or PBS alone as a control. The mice were euthanized 4 days post infection to harvest lung tissues and trachea tissues for viral load test (**d**) or histopathology evaluation (**e,f**). Data are presented as mean ± SD and representative of two independent experiments with similar results, n = 5

biologically independent animals. *P* values were determined by one-way ANOVA with Tukey's multiple comparison post-hoc test. Source data are provided as a Source Data file.

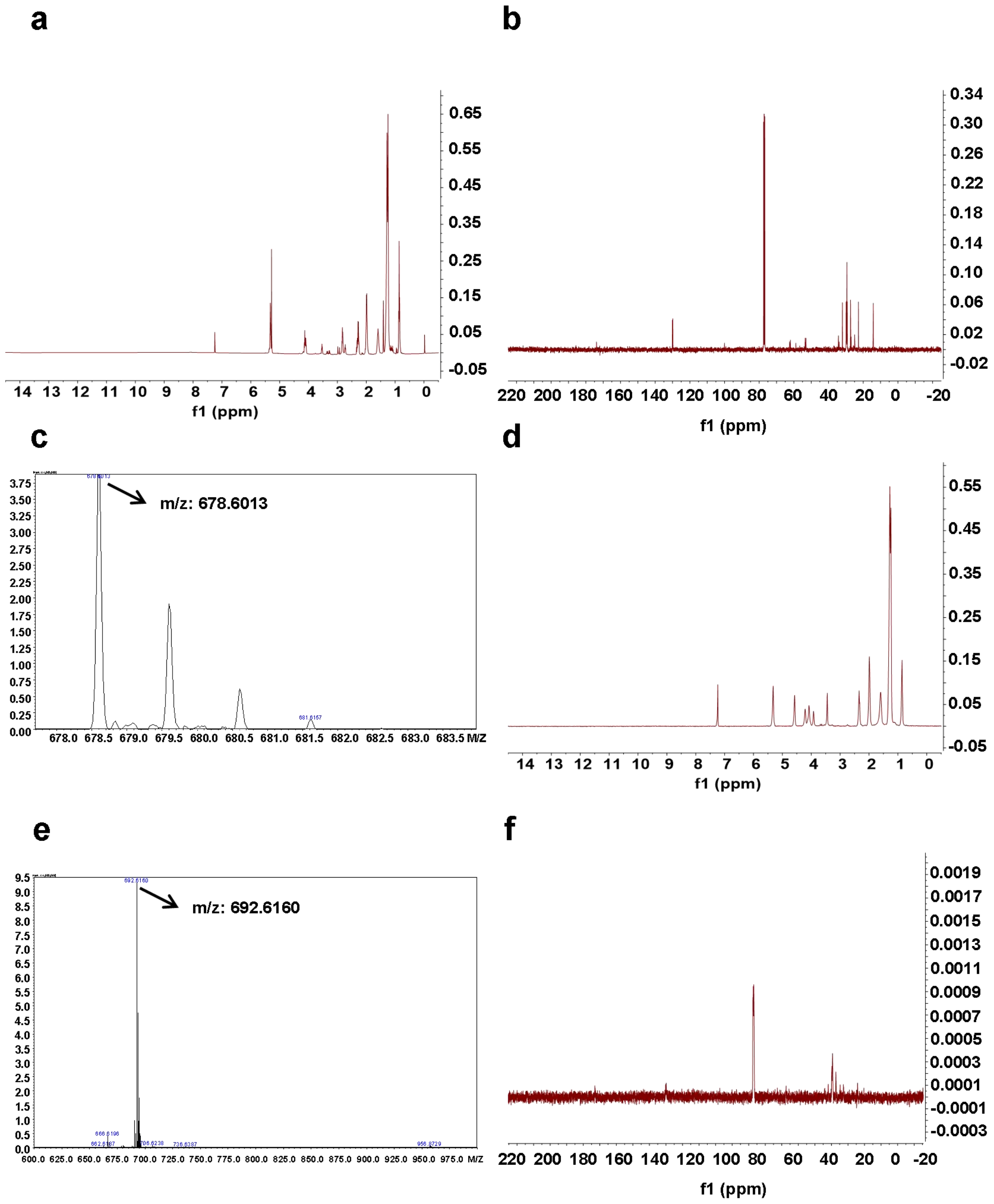

Supplementary Fig. 15 NMR spectra and MS results of ionizable lipid and targeting lipid. a, ^{1}H NMR spectrum (400 MHz, $CDCl_3$, room temperature) of ionizable lipid. **b,** ^{13}C NMR spectrum (100 MHz, $CDCl_3$, room temperature) of ionizable lipid. **c,** ESI IT-TOF MS spectrum of ionizable lipid. **d,** ^{1}H NMR spectrum (400 MHz, $CDCl_3$, room temperature) of targeting lipid. **e,** ESI IT-TOF MS spectrum of targeting lipid. **f,** ^{13}C NMR spectrum (100 MHz, $CDCl_3$, room temperature) of targeting lipid. Source data are provided as a Source Data file.

Supplementary Table 1. Single-cell PCR primer sequences

IgH	5'to3'
First PCR	
5′ L-VH 1	ACAGGTGCCCACTCCCAGGTGCAG
5′ L-VH 3	AAGGTGTCCAGTGTGARGTGCAG
5′ L-VH 4/6	CCCAGATGGGTCCTGTCCCAGGTGCAG
5′ L-VH 5	CAAGGAGTCTGTTCCGAGGTGCAG
3′ Cγ CH1	GGAAGGTGTGCACGCCGCTGGTC
Second PCR	
5′ VH1	CTAGTAGCAACTGCAACCGGTGTACATTCCCAGGTGCAGCTGGTGCAG
5′ VH1/5	CTAGTAGCAACTGCAACCGGTGTACATTCCGAGGTGCAGCTGGTGCAG
5′ VH3	CTAGTAGCAACTGCAACCGGTGTACATTCTGAGGTGCAGCTGGTGGAG
5′ VH3–23	CTAGTAGCAACTGCAACCGGTGTACATTCTGAGGTGCAGCTGTTGGAG
5′ VH4	CTAGTAGCAACTGCAACCGGTGTACATTCCCAGGTGCAGCTGCAGGAG
5′ 4–34	CTAGTAGCAACTGCAACCGGTGTACATTCCCAGGTGCAGCTACAGCAGTG
5′ VH 1–18	CTAGTAGCAACTGCAACCGGTGTACATTCCCAGGTTCAGCTGGTGCAG
5′ VH 1–24	CTAGTAGCAACTGCAACCGGTGTACATTCCCAGGTCCAGCTGGTACAG
5′ VH3–33	CTAGTAGCAACTGCAACCGGTGTACATTCTCAGGTGCAGCTGGTGGAG
5′ VH 3–9	CTAGTAGCAACTGCAACCGGTGTACATTCTGAAGTGCAGCTGGTGGAG
5′ VH4–39	CTAGTAGCAACTGCAACCGGTGTACATTCCCAGCTGCAGCTGCAGGAG

5′ VH 6–1	CTAGTAGCAACTGCAACCGGTGTACATTCCCAGGTACAGCTG CAGCAG
3′ JH 1/2/4/5	CCGATGGGCCCTTGGTCGACGCTGAGGAGACGGTGACCAG
3′ JH 3	CCGATGGGCCCTTGGTCGACGCTGAAGAGACGGTGACCATTG
3′ JH 6	CCGATGGGCCCTTGGTCGACGCTGAGGAGACGGTGACCGTG
Igκ	
First PCR	
5′ L Vκ 1/2	ATGAGGSTCCCYGCTCAGCTGCTGG
5′ L Vκ 3	CTCTTCCTCCTGCTACTCTGGCTCCCAG
5′ L Vκ 4	ATTTCTCTGTTGCTCTGGATCTCTG
3′ Cκ 543	GTTTCTCGTAGTCTGCTTTGCTCA
Second PCR	
5′Vκ 1–5	CAACCGGTGTACATTCAGAATTCGTACATTCTGACATCCAGAT GACCCAGTC
5′Vκ 1–9	CAACCGGTGTACATTCAGAATTCGTACATTCAGACATCCAGTT GACCCAGTCT
5′Vκ 1D–43	CAACCGGTGTACATTCAGAATTCGTACATTGTGCCATCCGGAT GACCCAGTC
5′Vκ 2–24	CAACCGGTGTACATTCAGAATTCGTACATGGGGATATTGTGAT GACCCAGAC
5′Vκ 2–28	CAACCGGTGTACATTCAGAATTCGTACATGGGGATATTGTGAT GACTCAGTC
5′Vκ 2–30	CAACCGGTGTACATTCAGAATTCGTACATGGGGATGTTGTGAT GACTCAGTC
5′Vκ 3–11	CAACCGGTGTACATTCAGAATTCGTACATTCAGAAATTGTGTT GACACAGTC
5′Vκ 3–15	CAACCGGTGTACATTCAGAATTCGTACATTCAGAAATAGTGAT GACGCAGTC
5′Vκ 3–20	CAACCGGTGTACATTCAGAATTCGTACATTCAGAAATTGTGTT GACGCAGTCT
5′Vκ 4–1	CAACCGGTGTACATTCAGAATTCGTACATTCGGACATCGTGAT

	GACCCAGT
3′Jκ 1/4	CAGCCACCGTACGCAGCTGTTTGATYTCCACCTTGGTC
3′Jκ 2	CAGCCACCGTACGCAGCTGTTTGATCTCCAGCTTGGTC
3′Jκ 3	CAGCCACCGTACGCAGCTGTTTGATATCCACTTTGGTC
3′Jκ 5	CAGCCACCGTACGCAGCTGTTTAATCTCCAGTCGTGTC

Igλ	5'to3'
First PCR	
5′ L Vλ 1	GGTCCTGGGCCCAGTCTGTGCTG
5′ L Vλ 2	GGTCCTGGGCCCAGTCTGCCCTG
5′ L Vλ 3	GCTCTGTGACCTCCTATGAGCTG
5′ L Vλ 4/5	GGTCTCTCTCSCAGCYTGTGCTG
5′ L Vλ 6	GTTCTTGGGCCAATTTTATGCTG
5′ L Vλ 7	GGTCCAATTCYCAGGCTGTGGTG
5′ L Vλ 8	GAGTGGATTCTCAGACTGTGGTG
3′ Cλ	CACCAGTGTGGCCTTGTTGGCTTG
Second PCR	
5′Vλ 1	CAACCGGTGTACATTCAGAATTCTCCTGGGCCCAGTCTGTGCT GACKCAG
5′Vλ 2	CAACCGGTGTACATTCAGAATTCTCCTGGGCCCAGTCTGCCCT GACTCAG
5′Vλ 3	CAACCGGTGTACATTCAGAATTCTCTGTGACCTCCTATGAGCT GACWCAG
5′Vλ 4/5	CAACCGGTGTACATTCAGAATTCTCTCTCTCSCAGCYTGTGCT GACTCA
5′Vλ 6	CAACCGGTGTACATTCAGAATTCTCTTGGGCCAATTTTATGCT GACTCAG
5′Vλ 7/8	CAACCGGTGTACATTCAGAATTCTCCAATTCYCAGRCTGTGGT GACYCAG
3′Cl	CAGCCACCGTACGCAGCTGGGYGGGAACAGAGTG

Supplementary Table 2 Characteristics of the selected donor of antibody 8-9D.

ID	Age (years)	Sex	Race	mAbs obtained at	ELISA Binding (titer)*		Pseudovirus neutralization (ID_{50})*		
					Anti-Spike IgG	Anti-RBD IgG	WT	Previous VOCs (GMTs)	Previous VOIs (GMTs)
#11	33	M	Asian	6 months after the second vaccination	2700	2700	84	68	45

M, male; GMTs, geometric mean titers. *Titers detected at 2 weeks after the second dose.

Supplementary Table 3 Molecular characteristics of neutralizing antibody 8-9D.

mAb	Heavy chain					Light chain			
	IGHV	IGHD	IGHJ	CDR3 length (aa)	SHM (%)	IGKV	IGKJ	CDR3 length (aa)	SHM (%)
**8-9D**	3-53*04	1-14*01, 2-15*01, 4-23*01	3*02	11	4.8	1-9*01	2*01	11	1.4

The program IGBLAST was used to analyze germline gene, germline divergence or degree of somatic hypermutation rate (SHM), the framework region (FR) and the loop length of CDR3 for each antibody clone. The CDR3 length was calculated in amino acids (aa).

Supplementary Table 4 Contacts between spike and 8-9D.

Van der Waals contacts[a]		Direct hydrogen bonds[b]		
Spike	8-9D	Spike	8-9D	Distance (Å)
ARG^{403}	SER^{L30}(2)	ARG^{403}[NH2]	SER^{L30}[OG]	3.4
*ASN^{405}	SER^{L93}(3)	*ASN^{405}[ND2]	SER^{L93}[OG]	2.9
THR^{415}	SER^{H57}(3), PHE^{H59}(6)	THR^{415}[OG1]	SER^{H57}[OG]	2.7
*ASN^{417}	TYR^{H34}(2), TYR^{H53}(4)	ASN^{417}[ND2]	TYR^{H34}[OH]	3.2
		ASN^{417}[ND2]	TYR^{H53}[OH]	3.7
		ASN^{417}[OD1]	TYR^{H53}[OH]	3.6
ASP^{420}	SER^{H57}(3)	ASP^{420}[OD2]	SER^{H57}[OG]	2.8
TYR^{421}	TYR^{H53}(1), PRO^{H54}(6), GLY^{H55}(3)	TYR^{421}[OH]	GLY^{H55}[N]	2.7
LEU^{455}	TYR^{H34}(6), SER^{H101}(1), GLY^{H102}(2)	LEU^{455}[O]	TYR^{H34}[OH]	2.5
PHE^{456}	ASP^{H99}(1), HIS^{H100}(2)			
ARG^{457}	PRO^{H54}(2)			
LYS^{458}	SER^{H32}(1)	LYS^{458}[NZ]	SER^{H32}[OG]	3.3
ASN^{460}	GLY^{H55}(1)			
TYR^{473}	SER^{H32}(3)	TYR^{473}[OH]	SER^{H32}[O]	3.6
ALA^{475}	ASN^{H33}(3)			
GLY^{476}	GLY^{H27}(1), LEU^{H28}(2), THR^{H29}(5)			
*ASN^{477}	THR^{H29}(2)	ASN^{477}[N]	THR^{H29}[OG1]	3.7
		ASN^{477}[OD1]	THR^{H29}[OG1]	3.3
ASN^{487}	ARG^{H98}(2)	ASN^{487}[OD1]	ARG^{H98}[NH1]	3.5
		ASN^{487}[OD1]	ARG^{H98}[NH2]	3.6
TYR^{489}	ASN^{H33}(1), ARG^{H98}(5), HIS^{H100}(1)			
GLN^{493}	SER^{H101}(6), TYR^{L32}(7)	GLN^{493}[NE2]	SER^{H101}[O]	2.3
SER^{494}	TYR^{L32}(6)	SER^{494}[N]	TYR^{L32}[OH]	3.7
		SER^{494}[OG]	TYR^{L32}[OH]	3.8

		SER^{494}[O]	TYR^{L32}[OH]	2.5
*ARG^{498}	SER^{L31}(1)	ARG^{498}[NH1]	SER^{L31}[OG]	3.0
*TYR^{501}	ILE^{L29}(5), SER^{L31}(1)	TYR^{501}[OH]	SER^{L31}[OG]	3.2
GLY^{502}	GLN^{L27}(2)			
VAL^{503}	GLN^{L27}(1)			
*HIS^{505}	SER^{L30}(2), HIS^{L90}(2)			

a: Van der Waals contacts have interatomic distance ≤ 4.0 Å and defined by CCP4i.

b: Putative H-bonds with distance cutoff 4 Å and angle range 90-270 degree defined by PISA.

*Stars indicate mutated residues from BA.5 strain comparing with original strain.

Supplementary Table 5. Cryo-EM data collection, refinement and validation statistics for 8-9D-Fab and Omicron BA.5 spike.

	8-9D-Fab - Spike	
**Data collection and processing**	Overall (3Fab-1Spike)	Local (1Fab-1RBD)
Voltage (kV)	300	
Defocus range (μm)	-1.5 to -2.0	
Pixel size (Å)	0.97	
Final particle images (no.)	296568	1610142
Map resolution (Å)	3.01	3.32
Map sharpening B factor ($Å^2$)	-123.5	-127.6
Initial model used	6XEY	Local density from overall map
Symmetry imposed	C3	C1
**Refinement**		
R.M.S. deviations		
Bond lengths (Å)	0.005	0.005
Bond angles (°)	0.957	0.978
MolProbity score	1.64	1.91
Clashscore	7.32	8.46
Rotamer outliers (%)	0.09	0.00
Ramachandran plot		
Favored (%)	96.44	92.80
Allowed (%)	3.56	7.20
Disallowed (%)	0.00	0.00
B factors ($Å^2$) min/max/mean		
Protein	30.00/167.22/68.51	30.00/93.32/62.63
Ligand	68.28/150.58/94.23	NA
