# Supplementary material for: A lung-selective delivery of mRNA encoding broadly neutralizing antibody against SARS-CoV-2 infection
Source: Nat Commun. 2023 Dec 5;14:8042. doi: 10.1038/s41467-023-43798-8 (PMC10697968; doi:10.1038/s41467-023-43798-8)
Supplement: Supplementary file 3 — Reporting Summary [file 41467_2023_43798_MOESM3_ESM.pdf]

nature portfolio

Corresponding author(s): Gong Cheng

Last updated by author(s): Nov 13, 2023

Reporting Summary

Nature Portfolio wishes to improve the reproducibility of the work that we publish. This form provides structure for consistency and transparency in reporting. For further information on Nature Portfolio policies, see our Editorial Policies and the Editorial Policy Checklist.

Statistics

For all statistical analyses, confirm that the following items are present in the figure legend, table legend, main text, or Methods section.

n/a	Confirmed	
☐	☒	The exact sample size (*n*) for each experimental group/condition, given as a discrete number and unit of measurement
☐	☒	A statement on whether measurements were taken from distinct samples or whether the same sample was measured repeatedly
☐	☒	The statistical test(s) used AND whether they are one- or two-sided<br>*Only common tests should be described solely by name; describe more complex techniques in the Methods section.*
☒	☐	A description of all covariates tested
☐	☒	A description of any assumptions or corrections, such as tests of normality and adjustment for multiple comparisons
☐	☒	A full description of the statistical parameters including central tendency (e.g. means) or other basic estimates (e.g. regression coefficient) AND variation (e.g. standard deviation) or associated estimates of uncertainty (e.g. confidence intervals)
☐	☒	For null hypothesis testing, the test statistic (e.g. *F*, *t*, *r*) with confidence intervals, effect sizes, degrees of freedom and *P* value noted<br>*Give P values as exact values whenever suitable.*
☒	☐	For Bayesian analysis, information on the choice of priors and Markov chain Monte Carlo settings
☒	☐	For hierarchical and complex designs, identification of the appropriate level for tests and full reporting of outcomes
☒	☐	Estimates of effect sizes (e.g. Cohen's *d*, Pearson's *r*), indicating how they were calculated

Our web collection on statistics for biologists contains articles on many of the points above.

Software and code

Policy information about availability of computer code

Data collection	ELISA and Neutralization data were collected using TECAN i-control 2.0. BLI binding data were collected by using Octet data acquisition v12.0. Flow cytometry data were collected using Sony ID7000™ software (v1.1.10) and Cell Sorter Software v3.1.1. Cryo-EM data were collected with SerialEM v 4.0.4. Indicators of kidney and liver injury were measured by Idexx VetTest 8008.
Data analysis	Flow cytometry data analysis was performed using FlowJo V10 and Cell Sorter Software v.3.1.1. Neutralization assays, viral titers and viral loads were analyzed using GraphPad Prism v.8.0. BLI binding data analysis was done by using FortéBio data analysis v12.1 and HT v12.1. Cryo-EM data processing was performed in MotionCor2 v.1.2.6, Gctf v.1.18, Gautomatch v.0.56, RELION v3.1 and cryoSPARC v3.3.2. Model building and refinement were performed using COOT v.0.9.6 and PHENIX v.1.19. Epitope was identified with CCP4i2 v1.0.2, Foldit Standalone and PISA. All structural figures were generated using ChimeraX v.1.3.

For manuscripts utilizing custom algorithms or software that are central to the research but not yet described in published literature, software must be made available to editors and reviewers. We strongly encourage code deposition in a community repository (e.g. GitHub). See the Nature Portfolio guidelines for submitting code & software for further information.

Data

Policy information about availability of data

All manuscripts must include a data availability statement. This statement should provide the following information, where applicable:

- Accession codes, unique identifiers, or web links for publicly available datasets
- A description of any restrictions on data availability
- For clinical datasets or third party data, please ensure that the statement adheres to our policy

The authors declare that all data supporting the results in this study are available in the paper and Supplementary Materials. The data generated in this study are provided in the Supplementary Information and Source Data file. The atomic coordinates and EM maps have been deposited into the Protein Data Bank (http://www.pdb.org) and the EM Data Bank, respectively: The whole complex of spike binding with three 8-9D Fabs (PDB: 8J1V [https://www.rcsb.org/structure/unreleased/8J1V], EMD: 35934 [https://www.ebi.ac.uk/emdb/EMD-35934]), local refined 8-9D Fab-RBD complex (PDB: 8J1T [https://www.rcsb.org/structure/unreleased/8J1T], EMD: 35932 [https://www.ebi.ac.uk/emdb/EMD-35932]). Sequences of the monoclonal antibody 8-9D characterized here has been deposited at GenBank (accession number: OQ868201 [https://www.ncbi.nlm.nih.gov/nuccore/OQ868201] and OQ868202 [https://www.ncbi.nlm.nih.gov/nuccore/OQ868202]).

Research involving human participants, their data, or biological material

Policy information about studies with human participants or human data. See also policy information about sex, gender (identity/presentation), and sexual orientation and race, ethnicity and racism.

Reporting on sex and gender	The donor of antibody 8-9D is male (biological attribute). Detailed Information about the participant has been provided in Supplementary Table 1.
Reporting on race, ethnicity, or other socially relevant groupings	The donor of antibody 8-9D is an Asian.
Population characteristics	The donor of antibody 8-9D is 33 years old. Detailed Information about the participant has been provided in supplementary Table 1.
Recruitment	Healthy volunteers who were planning to immunized with COVID-19 vaccines were recruited for blood donation. No potential selection bias was present.
Ethics oversight	The work was approved by the Institutional Review Board of Tsinghua University with an approval number of 20210061. Written informed consent was obtained from all participants.

Note that full information on the approval of the study protocol must also be provided in the manuscript.

Field-specific reporting

Please select the one below that is the best fit for your research. If you are not sure, read the appropriate sections before making your selection.

☒ Life sciences ☐ Behavioural & social sciences ☐ Ecological, evolutionary & environmental sciences

For a reference copy of the document with all sections, see nature.com/documents/nr-reporting-summary-flat.pdf

Life sciences study design

All studies must disclose on these points even when the disclosure is negative.

Sample size	Blood samples were obtained from 28 participants and all samples collected were analyzed. The sample size of plasma could reach statistical significance of antibody titers or Spike-specific memory B-cell percentages by two-tailed Wilcoxon signed-rank test. Mice number (n=3-5, each group) was selected to maintain the animals number n≥3/group throughout the study period considering the interim tissue collection (Zost, et al. Nature. 2020, 584, 443-449; Qiao, et al. Science. 2021, 371, 1374-1378). No statistical methods were used to predetermine the sample size.
Data exclusions	No data were excluded.
Replication	Experimental assays were performed in duplicate (or more) according to or exceeding standards in the field. All attempts at replication were successful. In all cases, representative figure displays were appropriately indicated.
Randomization	Animals were randomly allocated to the groups. For other experiments, randomization was not a relevant feature as we were applying a uniform set of techniques across a panel of plasma and antibodies.
Blinding	The investigators were blinded for lung pathology evaluation. For other experiments, blinding was not relevant as this is an observational study and we were applying a uniform set of measurements across the panel of plasma and antibodies.

Behavioural & social sciences study design

All studies must disclose on these points even when the disclosure is negative.

Study description	*Briefly describe the study type including whether data are quantitative, qualitative, or mixed-methods (e.g. qualitative cross-sectional, quantitative experimental, mixed-methods case study).*
Research sample	*State the research sample (e.g. Harvard university undergraduates, villagers in rural India) and provide relevant demographic information (e.g. age, sex) and indicate whether the sample is representative. Provide a rationale for the study sample chosen. For studies involving existing datasets, please describe the dataset and source.*
Sampling strategy	*Describe the sampling procedure (e.g. random, snowball, stratified, convenience). Describe the statistical methods that were used to predetermine sample size OR if no sample-size calculation was performed, describe how sample sizes were chosen and provide a rationale for why these sample sizes are sufficient. For qualitative data, please indicate whether data saturation was considered, and what criteria were used to decide that no further sampling was needed.*
Data collection	*Provide details about the data collection procedure, including the instruments or devices used to record the data (e.g. pen and paper, computer, eye tracker, video or audio equipment) whether anyone was present besides the participant(s) and the researcher, and whether the researcher was blind to experimental condition and/or the study hypothesis during data collection.*
Timing	*Indicate the start and stop dates of data collection. If there is a gap between collection periods, state the dates for each sample cohort.*
Data exclusions	*If no data were excluded from the analyses, state so OR if data were excluded, provide the exact number of exclusions and the rationale behind them, indicating whether exclusion criteria were pre-established.*
Non-participation	*State how many participants dropped out/declined participation and the reason(s) given OR provide response rate OR state that no participants dropped out/declined participation.*
Randomization	*If participants were not allocated into experimental groups, state so OR describe how participants were allocated to groups, and if allocation was not random, describe how covariates were controlled.*

Ecological, evolutionary & environmental sciences study design

All studies must disclose on these points even when the disclosure is negative.

Study description	*Briefly describe the study. For quantitative data include treatment factors and interactions, design structure (e.g. factorial, nested, hierarchical), nature and number of experimental units and replicates.*
Research sample	*Describe the research sample (e.g. a group of tagged Passer domesticus, all Stenocereus thurberi within Organ Pipe Cactus National Monument), and provide a rationale for the sample choice. When relevant, describe the organism taxa, source, sex, age range and any manipulations. State what population the sample is meant to represent when applicable. For studies involving existing datasets, describe the data and its source.*
Sampling strategy	*Note the sampling procedure. Describe the statistical methods that were used to predetermine sample size OR if no sample-size calculation was performed, describe how sample sizes were chosen and provide a rationale for why these sample sizes are sufficient.*
Data collection	*Describe the data collection procedure, including who recorded the data and how.*
Timing and spatial scale	*Indicate the start and stop dates of data collection, noting the frequency and periodicity of sampling and providing a rationale for these choices. If there is a gap between collection periods, state the dates for each sample cohort. Specify the spatial scale from which the data are taken*
Data exclusions	*If no data were excluded from the analyses, state so OR if data were excluded, describe the exclusions and the rationale behind them, indicating whether exclusion criteria were pre-established.*
Reproducibility	*Describe the measures taken to verify the reproducibility of experimental findings. For each experiment, note whether any attempts to repeat the experiment failed OR state that all attempts to repeat the experiment were successful.*
Randomization	*Describe how samples/organisms/participants were allocated into groups. If allocation was not random, describe how covariates were controlled. If this is not relevant to your study, explain why.*
Blinding	*Describe the extent of blinding used during data acquisition and analysis. If blinding was not possible, describe why OR explain why blinding was not relevant to your study.*

Did the study involve field work? ☐ Yes ☐ No

Field work, collection and transport

Field conditions	*Describe the study conditions for field work, providing relevant parameters (e.g. temperature, rainfall).*
Location	*State the location of the sampling or experiment, providing relevant parameters (e.g. latitude and longitude, elevation, water depth).*
Access & import/export	*Describe the efforts you have made to access habitats and to collect and import/export your samples in a responsible manner and in compliance with local, national and international laws, noting any permits that were obtained (give the name of the issuing authority, the date of issue, and any identifying information).*
Disturbance	*Describe any disturbance caused by the study and how it was minimized.*

Reporting for specific materials, systems and methods

We require information from authors about some types of materials, experimental systems and methods used in many studies. Here, indicate whether each material, system or method listed is relevant to your study. If you are not sure if a list item applies to your research, read the appropriate section before selecting a response.

Materials & experimental systems

n/a	Involved in the study	
☐	☒	Antibodies
☐	☒	Eukaryotic cell lines
☒	☐	Palaeontology and archaeology
☐	☒	Animals and other organisms
☒	☐	Clinical data
☒	☐	Dual use research of concern
☒	☐	Plants

Methods

n/a	Involved in the study	
☒	☐	ChIP-seq
☐	☒	Flow cytometry
☒	☐	MRI-based neuroimaging

Antibodies

Antibodies used

Goat Anti-Human IgG Fc (HRP)(Abcam, Cat#ab97225, 1:50,000 dilution),
Goat Anti-Human IgM mu chain (HRP) (Abcam, Cat#ab97205, 1:20,000 dilution),
FITC anti-human CD19 (Biolegend, Cat#302206, Clone HIB19, 1:100 dilution),
Pacific Blue™ anti-human CD3 (Biolegend, Cat#300431, Clone UCHT1, 1:100 dilution),
Pacific Blue™ anti-human CD8a (Biolegend, Cat#301023, Clone RPA-T8, 1:100 dilution),
Pacific Blue™ anti-human CD14 (Biolegend, Cat#325616, Clone HCD14, 1:100 dilution),
PerCP/Cyanine5.5 anti-human CD27 (Biolegend, Cat#356408, Clone M-T271, 1:100 dilution),
Ghost Dye™ UV 450 (Tonbo Biosciences Cat#: 13-0868-T500, 1:1000 dilution),
Alexa Fluor™ 700 anti-mouse CD45 (eBioscience, Cat# 56-0451-82, Clone 30-F11, 1:200 dilution),
Alexa Fluor® 488 anti-mouse CD11c (Biolegend, Cat# 117313, Clone N418, 1:200 dilution),
Pacific Blue™ anti-mouse/human CD11b (Biolegend, Cat#101223, Clone M1/70, 1:100 dilution) ,
Alexa Fluor® 647 anti-mouse I-A/I-E (Biolegend, Cat# 107618, Clone M5/114.15.2, 1:200 dilution),
PE/Cyanine7 anti-Mouse CD24 (Elabscience, Cat# E-AB-F1179UH, Clone M1/69, 1:100 dilution),
PE anti-Mouse CD64 (Elabscience, Cat# E-AB-F1186UD, Clone X54-5/7.1, 1:100 dilution),
HRP-labeled anti-Flag tag rabbit mAb (Proteintech, Cat. No# HRP-66008, 1:5,000 dilution),
HRP-labeled anti-V5 tag mouse mAb (GenScript, Cat. No# A01733, 1:3,000 dilution),
HRP-linked anti-human IgG (Invitrogen, Cat. No# A18817, 1:5,000 dilution),
anti-Flag tag rabbit antibody (Proteintech, Cat. No# 80010-1-RR, 1:500 dilution),
anti-V5 tag mouse mAb (Invitrogen, Cat. No# 37-7500, Clone 2F11F7, 1:1000 dilution),
Alexa Fluor 488 labeled anti-mouse IgG (H+L) cross-adsorbed secondary antibody (Invitrogen, Cat. No# A-11001, 1:500 dilution),
Alexa Fluor 594 labeled anti-rabbit IgG (H+L) secondary antibody (Invitrogen, Cat. No# A-21207, 1:500 dilution),
APC labeled anti-mouse CD31 Antibody (Biolegend, Cat. No#102409, Clone 390, 1:100 dilution),
APC labeled anti-mouse CD45 Antibody (Biolegend, Cat. No#157605, Clone QA17A26, 1:200 dilution),
APC labeled anti-mouse CD326 Antibody (Biolegend, Cat. No#118214, Clone G8.8, 1:200 dilution),
Monoclonal antibodies (CB6, C121, COV2-2130, and COVA1-16) are described in this paper.

Validation

Recombinant plasmids of monoclonal antibodies (CB6, C121, COV2-2130, and COVA1-16) were sequenced before transfection into HEK293F cell. Antibody specificity to RBD were validated by ELISA. Neutralizing activity of all the antibodies was verified by HIV-based pseudotyped virus assays. Details for all SARS-CoV-2 antibodies evaluated in this study is included in Figures, Supplementary data and Source data.
FITC anti-human CD19 (Biolegend, Cat#302206, Clone HIB19, 1:100 dilution) was validated by successful staining and flow cytometry

analysis according to the manufacturer's website https://www.biolegend.com/en-us/products/fitc-anti-human-cd19-antibody-717 and previous publication (Hagan T, et al. 2020. Cell. 178(6):1313-1328.e13.).
Pacific Blue™ anti-human CD3 (Biolegend, Cat#300431, Clone UCHT1, 1:100 dilution) was validated by successful staining and flow cytometry analysis according to the manufacturer's website https://www.biolegend.com/en-us/products/pacific-blue-anti-human-cd3-antibody-2846 and previous publication (Japp AS, et al. 2021. Cell. 184(3):827-839.e14.).
Pacific Blue™ anti-human CD8a (Biolegend, Cat#301023, Clone RPA-T8, 1:100 dilution) was validated by successful staining and flow cytometry analysis according to the manufacturer's website https://www.biolegend.com/en-us/products/pacific-blue-anti-human-cd8a-antibody-2851 and previous publication (Leclerc M, et al. 2019. Nat Commun. 10:3345.).
Pacific Blue™ anti-human CD14 (Biolegend, Cat#325616, Clone HCD14, 1:100 dilution) was validated by successful staining and flow cytometry analysis according to the manufacturer's website https://www.biolegend.com/en-us/products/pacific-blue-anti-human-cd14-antibody-3957 and previous publication (Crespo ÂC, et al. 2020. Cell. 182(5):1125-1139.).
PerCP/Cyanine5.5 anti-human CD27 (Biolegend, Cat#356408, Clone M-T271, 1:100 dilution) was validated by successful staining and flow cytometry analysis according to the manufacturer's website https://www.biolegend.com/en-us/products/percp-cyanine5-5-anti-human-cd27-antibody-8416 and previous publication (Sutton HJ, et al. 2021. Cell Reports. 34(6):108684.).
Ghost Dye™ UV 450 (Tonbo Biosciences Cat#: 13-0868-T500, 1:1000 dilution) was validated by successful staining and flow cytometry analysis according to the manufacturer's website https://cytekbio.com/products/ghost-dye-uv-450?variant=40581221187620 and previous publication (Mitchell JE, Lund MM, Starmer J, Ge K, Magnuson T, Shpargel KB, Whitmire JK. UTX promotes CD8+ T cell-mediated antiviral defenses but reduces T cell durability. Cell Rep. 2021 Apr 13;35(2):108966. doi: 10.1016/j.celrep.2021.108966. PMID: 33852868.)
Alexa Fluor™ 700 anti-mouse CD45 (eBioscience, Cat# 56-0451-82, Clone 30-F11, 1:200 dilution) was validated by successful staining and flow cytometry analysis according to the manufacturer's website https://www.thermofisher.cn/cn/zh/antibody/product/CD45-Antibody-clone-30-F11-Monoclonal/56-0451-82 and previous publication (Li Q. et al. 2018. Immunity. 48(2):258-270.e5.).
Alexa Fluor® 488 anti-mouse CD11c (Biolegend, Cat# 117313, Clone N418, 1:200 dilution) was validated by successful staining and flow cytometry analysis according to the manufacturer's website https://www.biolegend.com/en-us/products/alexa-fluor-488-anti-mouse-cd11c-antibody-2702 and previous publication (Lino AC et al. 2018. Immunity. 49(1):120-133. e9.).
Pacific Blue™ anti-mouse/human CD11b (Biolegend, Cat#101223, Clone M1/70, 1:100 dilution) was validated by successful staining and flow cytometry analysis according to the manufacturer's website https://www.biolegend.com/en-us/products/pacific-blue-anti-mouse-human-cd11b-antibody-3863 and previous publication (Zaman R, et al. 2021. Immunity. 54(9):2057-2071.e6.).
Alexa Fluor® 647 anti-mouse I-A/I-E (Biolegend, Cat# 107618, Clone M5/114.15.2, 1:200 dilution) was validated by successful staining and flow cytometry analysis according to the manufacturer's website https://www.biolegend.com/en-us/products/alexa-fluor-647-anti-mouse-i-a-i-e-antibody-3135 and previous publication (Uderhardt S, et al. 2019. Cell. 177(3):541-555.e17.).
PE/Cyanine7 anti-Mouse CD24 (Elabscience, Cat# E-AB-F1179UH, Clone M1/69, 1:100 dilution) was validated by successful staining and flow cytometry analysis according to the manufacturer's website https://www.elabscience.cn/ppe_cyanine7_anti_mouse_cd24_antibody_m1_69_e_ab_f1179uh-199035.html and previous publication (Rucci F, et al. 2010. Proc Natl Acad Sci USA. 107:3024.).
PE anti-Mouse CD64 (Elabscience, Cat# E-AB-F1186UD, Clone X54-5/7.1, 1:100 dilution) was validated by successful staining and flow cytometry analysis according to the manufacturer's website https://www.elabscience.cn/p-pe_anti_mouse_cd64_antibody_x54_5_7.1_e_ab_f1186ud-192517.html and previous publication (Ingersoll MA, et al. 2010. Blood 115: e10.).
HRP-labeled anti-Flag tag rabbit mAb (Proteintech, Cat. No# HRP-66008, 1:5,000 dilution) was validated by successful Western Blot staining according to the manufacturer's website https://www.ptglab.com/products/Flag-Tag-Antibody-HRP-66008.htm.
HRP-labeled anti-V5 tag mouse mAb (GenScript, Cat. No# A01733, 1:3,000 dilution) was validated by successful Western Blot staining according to the manufacturer's website https://www.genscript.com/antibody/A01733-THE_V5_Tag_Antibody_HRP_mAb_Mouse_.html.
HRP-linked anti-human IgG (Invitrogen, Cat. No# A18817, 1:5,000 dilution) was validated by successful ELISA according to the manufacturer's website https://www.thermofisher.cn/cn/zh/antibody/product/Goat-anti-Human-IgG-Fc-Secondary-Antibody-Polyclonal/A18817.
anti-Flag tag rabbit antibody (Proteintech, Cat. No# 80010-1-RR, 1:500 dilution) was validated by successful staining according to the manufacturer's website https://www.ptglab.com/products/DDDDK-tag-Antibody-80010-1-RR.htm.
anti-V5 tag mouse mAb (Invitrogen, Cat. No# 37-7500, Clone 2F11F7, 1:1000 dilution) was validated by successful staining according to the manufacturer's website https://www.thermofisher.com/antibody/product/V5-Tag-Antibody-clone-2F11F7-Monoclonal/37-7500.
Alexa Fluor 488 labeled anti-mouse IgG (H+L) cross-adsorbed secondary antibody (Invitrogen, Cat. No# A-11001, 1:500 dilution) was validated by successful staining according to the manufacturer's website https://www.thermofisher.cn/cn/zh/antibody/product/Goat-anti-Mouse-IgG-H-L-Cross-Adsorbed-Secondary-Antibody-Polyclonal/A-11001.
Alexa Fluor 594 labeled anti-rabbit IgG (H+L) secondary antibody (Invitrogen, Cat. No# A-21207, 1:500 dilution) was validated by successful staining according to the manufacturer's website https://www.thermofisher.cn/cn/zh/antibody/product/Donkey-anti-Rabbit-IgG-H-L-Highly-Cross-Adsorbed-Secondary-Antibody-Polyclonal/A-21207.
APC labeled anti-mouse CD31 Antibody (Biolegend, Cat. No#102409, Clone 390, 1:100 dilution) was validated by successful staining according to the manufacturer's website https://www.biolegend.com/en-gb/products/apc-anti-mouse-cd31-antibody-118.
APC labeled anti-mouse CD45 Antibody (Biolegend, Cat. No#157605, Clone QA17A26, 1:200 dilution) was validated by successful staining according to the manufacturer's website https://www.biolegend.com/en-gb/products/apc-anti-mouse-cd45-recombinant-antibody-18931.
APC labeled anti-mouse CD326 Antibody (Biolegend, Cat. No#118214, Clone G8.8, 1:200 dilution) was validated by successful staining according to the manufacturer's website https://www.biolegend.com/en-gb/products/apc-anti-mouse-cd326-ep-cam-antibody-4974.

Eukaryotic cell lines

Policy information about cell lines and Sex and Gender in Research

Cell line source(s)

HEK293T (ATCC, CRL3216),
HEK293F (Thermo Fisher Scientific, Cat#R79007),
HeLa-hACE2 (Prof. Qiang Ding, Tsinghua University; HeLa, ATCC CCL-2),

	Vero (ATCC, CCL81).
Authentication	All cell lines were frequently checked for cellular morphologies, growth rates and functions.
Mycoplasma contamination	All cell lines were tested and confirmed negative for Mycoplasma contamination.
Commonly misidentified lines (See ICLAC register)	No commonly misidentified cell lines were used in this study.

Palaeontology and Archaeology

Specimen provenance	*Provide provenance information for specimens and describe permits that were obtained for the work (including the name of the issuing authority, the date of issue, and any identifying information). Permits should encompass collection and, where applicable, export.*
Specimen deposition	*Indicate where the specimens have been deposited to permit free access by other researchers.*
Dating methods	*If new dates are provided, describe how they were obtained (e.g. collection, storage, sample pretreatment and measurement), where they were obtained (i.e. lab name), the calibration program and the protocol for quality assurance OR state that no new dates are provided.*

☐ Tick this box to confirm that the raw and calibrated dates are available in the paper or in Supplementary Information.

Ethics oversight	*Identify the organization(s) that approved or provided guidance on the study protocol, OR state that no ethical approval or guidance was required and explain why not.*

Note that full information on the approval of the study protocol must also be provided in the manuscript.

Animals and other research organisms

Policy information about studies involving animals; ARRIVE guidelines recommended for reporting animal research, and Sex and Gender in Research

Laboratory animals	For in vivo characterization of the organ-selective lipid nanoparticle systems for mAb mRNA, 6-8 weeks old C57BL/6 mice were purchased from Beijing Vital River Laboratory Animal Technology Co., Ltd. For viral challenge using authentic SARS-CoV-2, female four to six-week-old K18-hACE2 transgenic mice that were purchased from GemPharmatech (T037657) were used. Animals were housed in an negative pressured isolator under 12h light-dark cycles with temperature at 22 °C and humidity set points 50-60%.
Wild animals	This study did not involve wild animals.
Reporting on sex	Female mice were used for experiments.
Field-collected samples	The study did not involve samples collected from the field.
Ethics oversight	All animal care and experimental procedures were approved by the Institutional Animal Care and Use Committee (IACUC) of Shenzhen Bay Laboratory (BACG202101), and Changchun Veterinary Research Institute of Chinese Academy of Agricultural Sciences (11-2022-032) in accordance with the relevant guidelines for the protection of animal subjects.

Note that full information on the approval of the study protocol must also be provided in the manuscript.

Clinical data

Policy information about clinical studies

All manuscripts should comply with the ICMJEguidelines for publication of clinical research and a completedCONSORT checklist must be included with all submissions.

Clinical trial registration	*Provide the trial registration number from ClinicalTrials.gov or an equivalent agency.*
Study protocol	*Note where the full trial protocol can be accessed OR if not available, explain why.*
Data collection	*Describe the settings and locales of data collection, noting the time periods of recruitment and data collection.*
Outcomes	*Describe how you pre-defined primary and secondary outcome measures and how you assessed these measures.*

Dual use research of concern

Policy information about [dual use research of concern]

Hazards

Could the accidental, deliberate or reckless misuse of agents or technologies generated in the work, or the application of information presented in the manuscript, pose a threat to:

No	Yes	
☐	☐	Public health
☐	☐	National security
☐	☐	Crops and/or livestock
☐	☐	Ecosystems
☐	☐	Any other significant area

Experiments of concern

Does the work involve any of these experiments of concern:

No	Yes	
☐	☐	Demonstrate how to render a vaccine ineffective
☐	☐	Confer resistance to therapeutically useful antibiotics or antiviral agents
☐	☐	Enhance the virulence of a pathogen or render a nonpathogen virulent
☐	☐	Increase transmissibility of a pathogen
☐	☐	Alter the host range of a pathogen
☐	☐	Enable evasion of diagnostic/detection modalities
☐	☐	Enable the weaponization of a biological agent or toxin
☐	☐	Any other potentially harmful combination of experiments and agents

Plants

Seed stocks	*Report on the source of all seed stocks or other plant material used. If applicable, state the seed stock centre and catalogue number. If plant specimens were collected from the field, describe the collection location, date and sampling procedures.*
Novel plant genotypes	*Describe the methods by which all novel plant genotypes were produced. This includes those generated by transgenic approaches, gene editing, chemical/radiation-based mutagenesis and hybridization. For transgenic lines, describe the transformation method, the number of independent lines analyzed and the generation upon which experiments were performed. For gene-edited lines, describe the editor used, the endogenous sequence targeted for editing, the targeting guide RNA sequence (if applicable) and how the editor was applied.*
Authentication	*Describe any authentication procedures for each seed stock used or novel genotype generated. Describe any experiments used to assess the effect of a mutation and, where applicable, how potential secondary effects (e.g. second site T-DNA insertions, mosiacism, off-target gene editing) were examined.*

ChIP-seq

Data deposition

☐ Confirm that both raw and final processed data have been deposited in a public database such as GEO.

☐ Confirm that you have deposited or provided access to graph files (e.g. BED files) for the called peaks.

Data access links<br>*May remain private before publication.*	*For "Initial submission" or "Revised version" documents, provide reviewer access links. For your "Final submission" document, provide a link to the deposited data.*
Files in database submission	*Provide a list of all files available in the database submission.*
Genome browser session<br>(e.g. UCSC)	*Provide a link to an anonymized genome browser session for "Initial submission" and "Revised version" documents only, to enable peer review. Write "no longer applicable" for "Final submission" documents.*

Methodology

Replicates	*Describe the experimental replicates, specifying number, type and replicate agreement.*
Sequencing depth	*Describe the sequencing depth for each experiment, providing the total number of reads, uniquely mapped reads, length of reads and whether they were paired- or single-end.*
Antibodies	*Describe the antibodies used for the ChIP-seq experiments; as applicable, provide supplier name, catalog number, clone name, and lot number.*
Peak calling parameters	*Specify the command line program and parameters used for read mapping and peak calling, including the ChIP, control and index files used.*
Data quality	*Describe the methods used to ensure data quality in full detail, including how many peaks are at FDR 5% and above 5-fold enrichment.*
Software	*Describe the software used to collect and analyze the ChIP-seq data. For custom code that has been deposited into a community repository, provide accession details.*

Flow Cytometry

Plots

Confirm that:

- [x] The axis labels state the marker and fluorochrome used (e.g. CD4-FITC).
- [x] The axis scales are clearly visible. Include numbers along axes only for bottom left plot of group (a 'group' is an analysis of identical markers).
- [x] All plots are contour plots with outliers or pseudocolor plots.
- [x] A numerical value for number of cells or percentage (with statistics) is provided.

Methodology

Sample preparation	For SARS-CoV-2-specific memory B-cell analyses, PBMCs from vaccinated individuals were thawed and blocked with Human TruStain FcX Fc (Biolegend) for 10 minutes at 4 °C , followed by incubation in cell staining buffer (1× PBS, 2% FBS) with Biotinylated SARS-CoV-2 S protein (SPN-C82E9, Acro Biosystems), Biotinylated SARS-CoV-2 Spike Trimer (T19R, G142D, EF156-157del, R158G, L452R, T478K, D614G, P681R, D950N) (SPN-C82Ec, Acro Biosystems), or Biotinylated SARS-CoV-2 Spike Trimer (B.1.1.529/Omicron) (SPN-C82Ee, Acro Biosystems) for 60 min at 4 °C and then incubation for 30 min at 4 °C with the following anti-human antibodies (all at a 1:100 dilution): anti-CD19-FITC (Biolegend, 363008), anti-CD3-Pacific Blue (Biolegend, 300431), anti-CD8-Pacific Blue (Biolegend, 301023), anti-CD14-Pacific Blue (Biolegend, 325616), anti-CD27-PerCP/Cyanine5.5 (Biolegend, 356408), streptavidin-APC (Biolegend, 405207) and streptavidin-PE (Biolegend, 405203).<br>For RBD-specific single B-cell sorting, B cells were enriched among PBMCs from vaccinated individuals using a pan-B-cell isolation kit (Miltenyi Biotec, 130-101-638) according to the manufacturer's instructions. The enriched B cells were blocked with Human TruStain FcX Fc (Biolegend) for 10 minutes at 4 °C and incubated for 60 min at 4 °C in cell staining buffer (1× PBS, 2% FBS) with biotinylated SARS-CoV-2 spike RBD (Acro Biosystems, SPD-C82E9), and then incubated for 30 min at 4 °C with the following anti-human antibodies (all at a 1:100 dilution): anti-CD19-FITC (Biolegend, 363008), anti-CD3-Pacific Blue (Biolegend, 300431), anti-CD8-Pacific Blue (Biolegend, 301023), anti-CD14-Pacific Blue (Biolegend, 325616), anti-CD27-PerCP/Cyanine5.5 (Biolegend, 356408), streptavidin-APC (Biolegend, 405207) and streptavidin-PE (Biolegend, 405203).<br>For the pulmonary immune cell subset analysis, cells were isolated from enzymatically digested mouse lungs, and after the exclusion of doublets and debris, Single cell suspensions were stained with a LIVE/DEAD Ghost Dye™ UV 450 (Tonbo Biosciences, Cat. No# 13-0868-T500) to exclude dead cells from analysis, and then incubated with the antibody panel of CD45-AF700 (Invitrogen, Cat. No# 56-0451-82), CD11c-AF488 (Biolegend, Cat. No# 117313), CD11b-Pacific Blue (Biolegend, Cat. No# 101223), CD24-PE/Cyanine7 (Elabscience, Cat. No# E-AB-F1179UH), CD64-PE (Elabscience, Cat. No# E-AB-F1186UD), and I-A/I-E-AF647 (Biolegend, Cat. No# 107618) for 30 min at room temperature. For the analysis of cell types transfected in the lungs, 2 or 6 hours after intravenously injecting Lung-LNPs@mRNAeGFP, mice were sacrificed. Lungs were harvested and ground in the FACS buffer. The obtained solution was filtered using a 70 μm cell strainer and processed using red blood lysis buffer for 5 minutes. The samples were then centrifuged at 700 × g and subsequently incubated with a mixture, including Fixable Viability Dye eFluor™ 780, antibodies against epithelial (EpCAM, Biolegend, Cat. No# 118214), immune (CD45, Biolegend, Cat. No# 157605), and endothelial (CD31, Cat. No# 102409) cell markers at 4 °C or 30 minutes. The stained cells were washed twice with cold FACS buffer and resuspended in a 4% paraformaldehyde fix solution.
Instrument	MA900 Cell Sorter (Sony), Sony ID7000™ software (v1.1.10).
Software	FlowJo V10, Cell Sorter Software (v.3.1.1).
Cell population abundance	The percentages of memory B cells responding to the WT strain, Delta and Omicron BA.1 variant ranged from below 0.01 % to 0.65%. The proportion of SARS-CoV-2 RBD-specific B-cell among memory B-cell ranged from 0.02% to 0.26%.
Gating strategy	SARS-CoV-2 spike-binding memory B-cell was gated as CD3-CD8-CD14-CD19+CD27+Spike-PE+ and Spike-APC+. For SARS-CoV-2 RBD-specific antibody sorting, single CD3-CD8-CD14-CD19+CD27+RBD-PE+RBD-APC+ cells were gated. Gating schemes are shown in Extended Data Fig. 1. Alveolar macrophage cells were gated as CD45+CD11b- CD11c+ CD64+, CD103+DCs were gated as CD45+CD11c+ CD103+ CD24+, interstitial macrophages were gated as CD45+CD11b+ MHC II+ CD11c+ CD64+ CD24-,

CD11b+DCs were gated as CD45+CD11b+ MHC II+ CD11c+ CD24+ CD64-, and monocytes/macrophages was gated as CD45 +CD11b+ MHC II- CD64+/-. Granulocytes was gated as CD45+CD24+CD11c-. Gating schemes are shown in Extended Data Fig. 11. For the analysis of cell types transfected in the lungs, the stained cells were first gated for live single cells, followed by gating strategies for different cell types. Finally, the proportion of eGFP positive cells in each cell subtype was counted and statistically graphed.

☒ Tick this box to confirm that a figure exemplifying the gating strategy is provided in the Supplementary Information.

Magnetic resonance imaging

Experimental design

Design type	*Indicate task or resting state; event-related or block design.*
Design specifications	*Specify the number of blocks, trials or experimental units per session and/or subject, and specify the length of each trial or block (if trials are blocked) and interval between trials.*
Behavioral performance measures	*State number and/or type of variables recorded (e.g. correct button press, response time) and what statistics were used to establish that the subjects were performing the task as expected (e.g. mean, range, and/or standard deviation across subjects).*

Acquisition

Imaging type(s)	*Specify: functional, structural, diffusion, perfusion.*
Field strength	*Specify in Tesla*
Sequence & imaging parameters	*Specify the pulse sequence type (gradient echo, spin echo, etc.), imaging type (EPI, spiral, etc.), field of view, matrix size, slice thickness, orientation and TE/TR/flip angle.*
Area of acquisition	*State whether a whole brain scan was used OR define the area of acquisition, describing how the region was determined.*

Diffusion MRI ☐ Used ☐ Not used

Preprocessing

Preprocessing software	*Provide detail on software version and revision number and on specific parameters (model/functions, brain extraction, segmentation, smoothing kernel size, etc.).*
Normalization	*If data were normalized/standardized, describe the approach(es): specify linear or non-linear and define image types used for transformation OR indicate that data were not normalized and explain rationale for lack of normalization.*
Normalization template	*Describe the template used for normalization/transformation, specifying subject space or group standardized space (e.g. original Talairach, MNI305, ICBM152) OR indicate that the data were not normalized.*
Noise and artifact removal	*Describe your procedure(s) for artifact and structured noise removal, specifying motion parameters, tissue signals and physiological signals (heart rate, respiration).*
Volume censoring	*Define your software and/or method and criteria for volume censoring, and state the extent of such censoring.*

Statistical modeling & inference

Model type and settings	*Specify type (mass univariate, multivariate, RSA, predictive, etc.) and describe essential details of the model at the first and second levels (e.g. fixed, random or mixed effects; drift or auto-correlation).*
Effect(s) tested	*Define precise effect in terms of the task or stimulus conditions instead of psychological concepts and indicate whether ANOVA or factorial designs were used.*

Specify type of analysis: ☐ Whole brain ☐ ROI-based ☐ Both

Statistic type for inference (See Eklund et al. 2016)	*Specify voxel-wise or cluster-wise and report all relevant parameters for cluster-wise methods.*
Correction	*Describe the type of correction and how it is obtained for multiple comparisons (e.g. FWE, FDR, permutation or Monte Carlo).*

Models & analysis

n/a	Involved in the study
☐	☐ Functional and/or effective connectivity
☐	☐ Graph analysis
☐	☐ Multivariate modeling or predictive analysis

Functional and/or effective connectivity	*Report the measures of dependence used and the model details (e.g. Pearson correlation, partial correlation, mutual information).*
Graph analysis	*Report the dependent variable and connectivity measure, specifying weighted graph or binarized graph, subject- or group-level, and the global and/or node summaries used (e.g. clustering coefficient, efficiency, etc.).*
Multivariate modeling and predictive analysis	*Specify independent variables, features extraction and dimension reduction, model, training and evaluation metrics.*
